# Plasmid Costs Explain Plasmid Maintenance, Irrespective of the Nature of Compensatory Mutations

**DOI:** 10.3390/antibiotics12050841

**Published:** 2023-05-01

**Authors:** João S. Rebelo, Célia P. F. Domingues, Francisco Dionisio

**Affiliations:** 1 cE3c—Centre for Ecology, Evolution and Environmental Changes & CHANGE, Global Change and Sustainability Institute, Faculdade de Ciências, Universidade de Lisboa, 1749-016 Lisboa, Portugal; joaorebelo_4@hotmail.com (J.S.R.); celiapfd@hotmail.com (C.P.F.D.); 2 INIAV—National Institute for Agrarian and Veterinary Research, 2780-157 Oeiras, Portugal

**Keywords:** conjugative plasmids, antibiotic resistance, conjugation, fitness cost, adaptation, computer simulations

## Abstract

Conjugative plasmids often carry virulence and antibiotic-resistant genes. Therefore, understanding the behavior of these extra-chromosomal DNA elements gives insights into their spread. Bacteria frequently replicate slower after plasmids’ entry, an observation inconsistent with the plasmids’ ubiquity in nature. Several hypotheses explain the maintenance of plasmids among bacterial communities. However, the numerous combinations of bacterial species and strains, plasmids, and environments claim a robust elucidatory mechanism of plasmid maintenance. Previous works have shown that donor cells already adapted to the plasmid may use the plasmid as a ‘weapon’ to compete with non-adapted plasmid-free cells. Computer simulations corroborated this hypothesis with a wide range of parameters. Here we show that donor cells benefit from harboring conjugative plasmids even if compensatory mutations in transconjugant cells occur in the plasmid, not on chromosomes. The advantage’s leading causes are as follows: mutations take time to appear, many plasmids remain costly, and re-transfer of mutated plasmids usually occurs in sites distant to the original donors, implying little competition between these cells. Research in previous decades cautioned against uncritical acceptance of the hypothesis that resistance cost helps to preserve antibiotics’ effectiveness. This work gives a new twist to this conclusion by showing that costs help antibiotic-resistant bacteria to compete with plasmid-free cells even if compensatory mutations appear in plasmids.

## 1. Introduction

Conjugative plasmids are mobile DNA elements that significantly drive horizontal gene transfer among bacteria. Their ability to transfer across taxa is relevant from the public health perspective because they frequently carry antibiotic resistance and virulence genes. As such, they have been responsible for the emergence of multidrug resistance in clinical pathogens [1,2,3].

The striking ability of conjugative plasmids to move between bacterial species and strains implies that even if a plasmid and its current host have already adapted to each other, it may be costly to the new one [4]. We can envisage at least three main reasons for that. First, the chromosome of the two species is different, which means that the interaction of the plasmid genes with the other genes differs in the two cells. Even if the two hosts (the original and the new one) are relatively similar at the protein level, the two bacterial species sometimes exhibit a differential regulation of homologous genes [5,6]. Indeed, if codon usage differs in the recipient cell, translation of plasmid genes may become costly to the new host [7] (but see [8]). Secondly, the two strains or species may contain mobile genetic elements, e.g., other plasmids. Again, there are interactions between the genes of a focal plasmid and the genes of plasmids or other mobile genetic elements present in the two different cells, and these interactions may implicate the focal plasmid’s behavior [9,10,11,12]. Thirdly, the two species occupy different niches. For example, when a conjugative plasmid transfers between *S. enterica* serovar Typhimurium to *E. coli* K12, it moves from a human pathogen to a commensal. 

Possibly, the plasmid carries genes for antibiotic resistance, virulence, bacteriocins, or other valuable genes that would compensate for the plasmid fitness cost. However, these genes are only beneficial in a few situations, not always. For example, resistance genes are not helpful if the environment does not contain antibiotics. Moreover, even if these genes are useful to the host, its chromosome may recruit these genes and discard the plasmid [13,14]. Furthermore, one may assume that plasmids have opportunities to incorporate those advantageous genes only after securing their success, including spreading to many hosts. For example, antibiotic-resistant conjugative plasmids of the 1980s were similar to those observed in bacteria isolated during the pre-antibiotic era but without resistance genes [15,16].

With so many possible combinations of conjugative plasmids, bacterial hosts, and environments, one must find a robust and explanatory mechanism to explain plasmid success, and that mechanism must be independent of putative beneficial genes or mutations. Recently, we proposed a mechanism that relies precisely on the plasmid fitness costs. According to this mechanism, plasmids may act as weapons of the donor cells to which they are already adapted if bacteria grow and transfer plasmids in structured habitats [17]. 

A mathematical model and thousands of simulations with different combinations of parameters (plasmid transfer rate, the cost to the transconjugant cell before and after the appearance of compensatory mutations, and the number of generations needed for the rise of compensatory mutations) have shown that the evolutionary success of donor cells is higher when comparing with the similar system without plasmid transfer (with the same parameters) [17]. This work further investigates the hypothesis that plasmid-bearing cells can use their plasmids as biological weapons.

In our previous research, we assumed that compensatory mutations would occur in the chromosomes of transconjugants and descendants [17]. However, here we investigated the evolutionary success of the donor population if compensatory mutations in transconjugants occur in the plasmid rather than in the chromosome. We aim to explore the effect of this on the donor population’s success.

We hypothesize that if the harm caused by conjugative plasmids on recipient cells is the reason for the higher success of donor cells, then the plasmid should remain advantageous even if compensatory mutations occur in the plasmid [17]. However, when plasmids contain mutations that compensate entirely for their cost, they become neutral in the second-order transconjugants (cells that received the plasmid from transconjugants), third-order transconjugants, and so on. As a result, the plasmid would cause less harm to the local community of bacterial cells, thereby potentially undermining the success of the donors.

An earlier study by Zwanzig et al. has shown that plasmids have higher odds of survival if compensatory mutations occur in them rather than in chromosomes. However, that study focused on a liquid (unstructured) environment [18]. In these environments, every bacteria in the medium can use resources unused by slow-growing transconjugants rather than just the donating cell and a few others nearby as in structured habitats. 

If the plasmid harbors costly genes of virulence or antibiotic resistance, plasmid-bearing bacteria already adapted to the plasmid gain an even higher competitive advantage when competing in structured habitats. After transfer to other bacteria, plasmid–bacteria dyads may adapt to each other. However, we show here that plasmids confer an advantage to the original, adapted population, even if compensatory mutations occur in plasmids. We further show that the benefit is similar to the cases where compensatory mutations occur in chromosomes in most parameters’ combinations. This work uses a computer model of interacting donor, recipient, and transconjugant cells in a structured habitat. In these simulations, bacteria replicate, transfer or receive plasmids or die; everything happens in a structured habitat. Krone, Top, and colleagues experimentally validated these models in previous studies [19,20,21]. The arena where bacteria interact and replicate contains 1000 × 1000 sites. The size of each bacterial cell is of the order of ~1 μm, so these 10^6^ sites correspond to about 1 mm^2^. Each simulation performs an average of 1000 bacterial generations. 

## 2. Results

### 2.1. Do Donors Benefit from Plasmids with Compensatory Mutations in Plasmids?

To test whether donors have an increased fitness due to plasmid transfers even if compensatory mutations occur in the plasmid (and not in the chromosome), we compared the success of the donor population with and without plasmid transfer and, in the former case, with adaptive mutations in plasmids. If donors have increased fitness, we compared that increase with the one observed in the system where compensatory mutations occur in chromosomes (not plasmids). 

We calculated the fitness of the donors’ population using Equation (3) in the Materials and Methods section. In Equation (3), type A bacteria represent the initial donor cells and their descendants. In the same Equation (3), type B bacteria represent the sum of recipients and their descendants, as well as transconjugants (and descendants) and segregants (and descendants). The main objective of this paper is to compare the change in the success of the donors due to plasmid transfer in two scenarios: compensatory mutations occurring in the chromosome versus plasmid. 

In 141/144 conditions, the qualitative conclusion was similar in the two cases: for a specific parameters’ combination, if the donor population increases, decreases, or does not change its fitness significantly by donating the plasmid to other cells in the system where compensatory mutations occur in the chromosome, then the donor population increases, decreases, or does not change its fitness significantly in the system where compensatory mutations occur in plasmids. In the remaining three parameters’ combinations, all for the adaptation period of 70 generations and b = 0.2, the qualitative conclusions did not match. In two combinations (c = 0.1, γ*_max_* = 0.1, D/(D + R) = 1%; and c = 0, γ*_max_* = 1, D/(D + R) = 99%), the fitness of the donor population did not change significantly by donating the plasmid in the system where compensatory mutations occur in the chromosome but increased significantly in the system where the compensatory mutation occurred in the plasmid. In the last combination (c = 0, γ*_max_* = 0.01, D/(D + R) = 0.1%), the opposite occurred; namely, the fitness of the donor population increased by donating the plasmid in the system where compensatory mutations occurred in the chromosome but did not change significantly in the system where the compensatory mutation occurred in the plasmid. 

**Figure 1:**
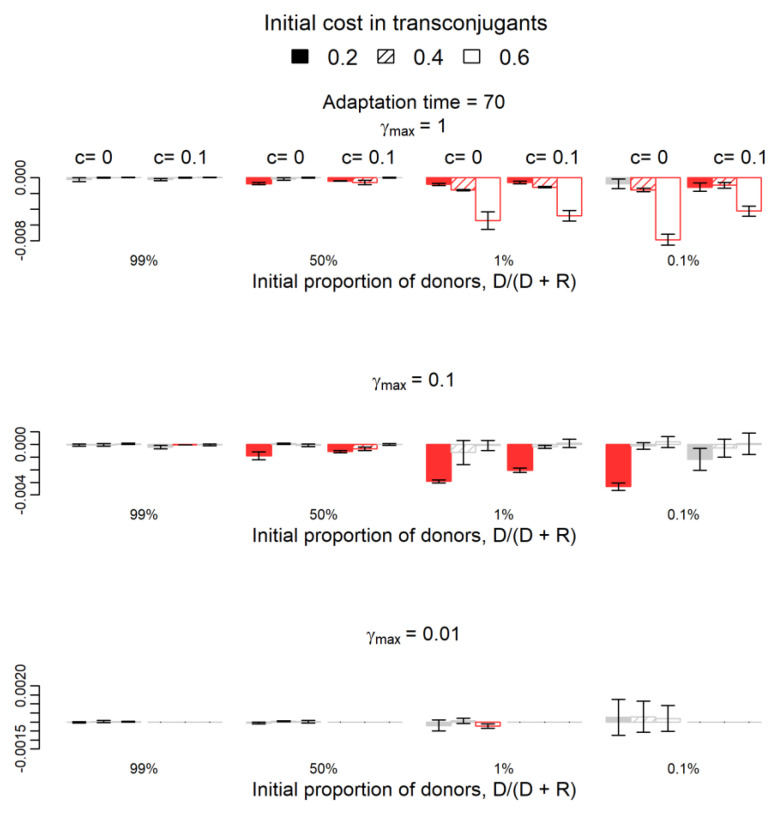
Variation of the relative fitness of donors if compensatory mutations occur on the plasmid instead of the chromosome and after 70 generations. The 22 red columns are the cases where variations (negative variations in all cases) were statistically significant (one-sample *t*-test, *p*-value < 0.05), while the grey columns represent the 50 cases where the difference is not significantly different from zero (*p*-value > 0.05). Red columns (negative values) show the cases where the success of donors is significantly lower if compensatory mutations occur on the plasmid. The parameter c (0 or 0.1) represents the fitness cost of the plasmid in donor cells or the adapted transconjugants. Filling indicates the parameter b, the initial cost in transconjugants: 0.2 (filled), 0.4 (striped), and 0.6 (empty). Parameter γ*_max_* represents the maximum plasmid transfer rate from plasmid-bearing cells to plasmid-free cells (recipients or segregants). For γ*_max_* = 0.01 and c = 0.1, donor cells disappear in both simulations, so the respective columns are not represented.

**Figure 2:**
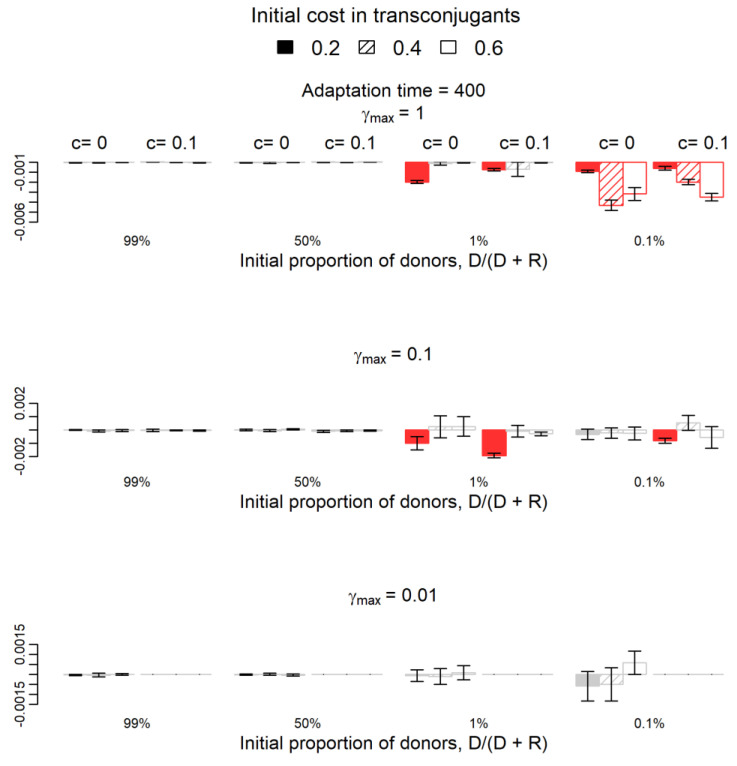
Variation of the relative fitness of donors if compensatory mutations occur on the plasmid instead of the chromosome and after 400 generations. The 11 red columns are the cases where variations (negative variations in all cases) were statistically significant (one-sample *t*-test, *p*-value < 0.05), while the grey columns represent the 61 cases where the difference is not significantly different from zero (*p*-value > 0.05). Red columns (negative values) show the cases where the success of donors is significantly lower if compensatory mutations occur on the plasmid. The parameter c (0 or 0.1) represents the fitness cost of the plasmid in donor cells or the adapted transconjugants. Filling indicates the parameter b, the initial cost in transconjugants: 0.2 (filled), 0.4 (striped), and 0.6 (empty). Parameter γ*_max_* represents the maximum plasmid transfer rate from plasmid-bearing cells to plasmid-free cells (recipients or segregants). For γ*_max_* = 0.01 and c = 0.1, donor cells disappear in both simulations, so the respective columns are not represented.

We now proceed with a qualitative analysis of the differences between the two systems (compensatory mutations appearing in plasmid versus chromosome). Figure 1 presents the results if compensatory mutations appear after 70 generations and Figure 2 if they appear after 400 generations. 

The fitness of the donor population in the system where compensatory mutations occur in plasmids is not statistically significantly different from that in the system where compensatory mutations occur in chromosomes in 111/144 cases (*p*-value > 0.05, grey columns in Figure 1 and Figure 2). Columns in red appear in parameters’ combinations where the donor population’s fitness was statistically significantly lower in the system where compensatory mutations occur in plasmids than in the system where these mutations occur in chromosomes. As expected, the increase of donors’ fitness due to plasmid transfer was higher in the system where mutations occur in the chromosome in many more parameters’ combinations (33/144 in red in the two Figures) than the opposite (zero cases out of 144 combinations). Most of the 33 (red) cases occur when the initial frequency of donors is low for both adaptation periods and when the initial frequency of donors is 50% for the shorter adaptation period of 70 generations. These results hold only for higher conjugation rates $\gamma_{max}\geq 0.1$ (Figure 1 and Figure 2).

### 2.2. How Does Plasmid Fitness Compare When Mutations Occur in Plasmids versus Chromosomes?

We then compared the plasmid’s fitness (i.e., the fitness of donors + transconjugants relative to plasmid-free cells) in simulations with compensatory mutations in the plasmid versus the chromosome. In 136/144 of the parameter combinations, the differences were not significant (Student *t*-test, *p* > 0.05), i.e., the fitness of the plasmids is not different if compensatory mutations occur in the plasmids or chromosomes. In only 7/144 parameter combinations, the fitness of the plasmids was higher in simulations where compensatory mutations occur in plasmids than in simulations where mutations occur in chromosomes. In 1/144 cases, we observed the opposite.

## 3. Discussion

Our results show that donor cells gain an advantage for harboring the plasmid in most parameters’ combinations, even when compensatory mutations appear in the plasmid. This result was unexpected because conjugative plasmids become neutral in future hosts if compensatory mutations appear in them (in contrast, plasmids remain costly for tens or hundreds of bacterial generations every time plasmids transfer into new recipient cells until compensatory mutations arise if compensatory mutations occur in chromosomes). Therefore, our initial expectation was that the positive impact on donor cells observed in the system where compensatory mutations occur in chromosomes might strongly fade or even disappear in the system where these mutations occur in plasmids. 

There are three main reasons for our results. First, only after tens or hundreds of generations do these mutations occur. Secondly, after many generations, mutated plasmids rise in sites relatively away from the original donor cells, decreasing the opportunities for competition between the two cell types. Thirdly, even if some plasmids receive compensatory mutations, the other transconjugants still harbor the costly plasmid.

Furthermore, only in 33/144 cases, donors’ success is lower when compensatory mutations appear in plasmids than when it appears in chromosomes. Another relevant result is that the plasmids’ fitness is not different if compensatory mutations occur in the plasmids or chromosomes in 94% (=136/144) of combinations. These and previous results [17,22] tell us that the primary selective force maintaining conjugative plasmids among bacterial communities is their positive impact on donors in structured habitats. 

It is important to stress that most bacteria live in structured habitats where social interactions may play an essential role in microbial evolution [23,24]. In our previous work, we derived a mathematical condition for the plasmid to give an advantage to donor cells over recipient cells at the beginning of simulations (when there are still no transconjugants) [17]. Upon analyzing social interactions between bacteria [25,26], a simple mathematical expression defined the condition under which donors benefit from plasmid donation. The condition is D/R > c/b, where D and R are the numbers of donor and recipient cells near a focal donor cell, and the parameters c and b represent the plasmid fitness cost of the adapted and non-adapted plasmid, respectively [17]. The replicon where the compensatory mutations appear (plasmid versus chromosome) was not an issue in its derivation because the focus was on a period where those mutations were still absent. In this sense, the results presented here agree with our expectations.

On the other hand, there is clonal competition between transconjugants harboring the adapted plasmid and donor cells (and their descendants). This competition occurs because transconjugant cells harboring the adapted plasmid have an advantage over cells containing the non-adapted plasmid, hence diminishing the relative advantage of the original donor cells. However, the results presented here show that, even after 1000 generations, donors benefit from plasmid donation, irrespectively of the replicon where compensatory mutations occur, in most parameters’ combinations. Therefore, the plasmid-mediated social interaction between donors and recipients is a dominant force in selecting plasmid transfer ability rather than the parasitic behavior of plasmids. 

In a previous article, Zwanzig et al. [18] showed with a mathematical model and computer simulations that plasmids have higher fitness if compensatory mutations appear in the plasmid rather than in the chromosome. This conclusion resulted from the analysis of ordinary differential equations and simulations where the assumption is that bacteria interact with each other with equal probability. That is the case of unstructured environments such as liquid media with agitation. Thus, if a bacterium does not use its available resources, any other cell can use them, not just a minority of cells. In particular, if a bacterium uses fewer resources because it has received a plasmid from a donor cell, all other bacteria in that environment can use the resources, not just the donor cell. 

However, this paper concerns structured habitats, where the fitness effects of interactions between cells only impact nearby cells. Therefore, we checked if the conclusions by Zwanzig et al. [18] hold in these environments. Only in 7 out of 144 cases, the fitness of the plasmids was higher in simulations where compensatory mutations occur in plasmids than in simulations where mutations occur in chromosomes. In 136/144 cases, whether compensatory mutations occur in the plasmids or chromosomes was irrelevant to the evolutionary success of plasmids. 

Over the past three decades, microbiologists have shown that there is no point in halting the consumption of certain antibiotics hoping to give a fitness advantage to antibiotic-sensitive bacteria and increase the effectiveness of antibiotics [27]. The reason is that compensatory mutations appear relatively fast. This paper and our previous works [17,22] have shown that not only is it useless to follow that strategy, but it is the plasmid cost conferred to transconjugants that maintain antibiotic-resistant bacteria, including pathogens. Therefore, our results suggest that conjugative plasmids will continue contributing to the striking number of deaths, close to five million per year, associated with antibiotic resistance worldwide [28,29]. Either one avoids using antibiotics immediately after their development, for example, understanding which factors impact antibiotic use [30,31], or one finds new strategies to combat the rise of antibiotic resistance [32,33].

This study has several limitations. The most important limitation is that we performed this study solely with computer models and theoretical arguments. However, we used a computer model previously tested with controlled experiments [19,20,21]. Specific “real” examples may matter; a recent study of the host adaption of the artificial plasmids pBR322 to its host has shown that mutations in plasmids could help the maintenance of the plasmid in bacterial cells under stress; however, these mutations may impose a high burden to the host. The result of this burden is that some compensatory mutations occurring in the chromosome counter-selected the mutations that occurred in the plasmid [34]. On the other hand, our computer simulations have an advantage over typical laboratory experiments: by testing many combinations of parameters, we are testing the hypothesis with many different types of bacteria, plasmids, and growth conditions. 

## 4. Materials and Methods

As explained in the Introduction section, we used a computer model of interacting bacterial cells: plasmid-bearing cells (the original donors or the transconjugant cells) and recipient cells. These cells replicate, die, or interact through plasmid transfer in a structured habitat containing 1000 × 1000 sites, corresponding to an area of about 1 mm^2^. These computational models were developed in [19,20,21]. Each simulation performs an average of 1000 bacterial generations. If conjugation occurs, the cell that receives the plasmid (a transconjugant cell) has a fitness cost b for acquiring the plasmid; this cost may take the values 0.2, 0.4 or 0.6, corresponding to a cost of 20%, 40% or 60%. In this paper, we denote replicated transconjugants also as transconjugants. After a certain number of duplications (70 or 400), we assume that the bacterium has a compensatory mutation, and consequently, the cost reduces to the cost paid by donor cells, c. The cost c may take the values 0 or 0.1. The conjugation rate γ*_max_* may also take three different values, 1, 0.1, or 0.01. Cells can replicate if there are empty sites around them. When bacteria already occupy 95% of these 10^6^ sites, the simulation eliminates many of them, keeping only 50% alive. Then, the cycle starts again, with plasmid transfers (with a certain probability if a donor or a transconjugant and recipient cells are close to each other) and bacteria replication. With two values for parameter c, three values for b, three for γ*_max_*, four initial frequencies of donor cells, and two adaptation periods, we analyzed a total of 2 × 3 × 3 × 4 × 2 = 144 conditions to calculate the fitness of the donor population and compared the two cases where compensatory mutations occur in chromosome versus plasmids. 

### 4.1. The Computational Model

The model was developed in Python programming language and is available on GitHub (https://github.com/jrebelo27/harmfull_plasmids_compensatory_mutation_in_plasmid, accessed on 25 April 2023). This model is an adaptation of our previously proposed model, which, in turn, followed the same parameters experimentally tested by Krone’s and Top’s research groups [19,20,21]. We now explain the computational model in detail following the ODD (Overview, Design Concept, Details) protocol to describe our individual-based model [35,36,37].

#### 4.1.1. Purpose

This model aims to analyze the effect of compensatory mutation occurring in the plasmid under the social model previously proposed to explain plasmid maintenance [17]. The model will simulate the structured environment and test the importance of the frequency of plasmid donor bacteria relative to recipient bacteria, the conjugation rate, and the costs associated with the plasmid. 

#### 4.1.2. Entities, State Variables, and Scales

In this model, we consider the following entities: empty spaces and bacteria.

To characterize the empty spaces, we consider the x-coordinate and the y-coordinate. These coordinates can assume values between 1 and 1000 and represent the location of the bacteria on the grid. We consider that the grid has periodic boundaries—that is, the upper margin is linked to the lower margin, and the left margin is linked to the right margin. To characterize the bacteria, we also consider their x-coordinate and y-coordinate. Furthermore, we equally consider:

- Type, which ranges from 1 to 6—defines the type of bacteria, recipient (1), donor (2), transconjugant (3), adapted transconjugant that lost the plasmid (4), non-adapted transconjugant that lost the plasmid (5), or donor that lost the plasmid (6);
- Permanent plasmid fitness cost, which can be 0 or 0.1—this is the plasmid cost for donors and transconjugants carrying the plasmid already adapted;
- Initial plasmid fitness cost, which may be 0.2, 0.4, or 0.6—this is the plasmid cost for transconjugants carrying the non-adapted plasmid;
- Adaptation time, which can be 70 or 400—represents the number of times the plasmid is in a transconjugant bacterium that divides until its cost decreases;
- Local neighborhood, 3 × 3 space centered on the bacterium—these are the spaces into which the bacterium can duplicate or where another bacterium can be present to receive a plasmid through conjugation;
- Nutrient neighborhood, 7 × 7 space centered on the bacteria—allows defining the number of nutrients available, given by the proportion of empty spaces in these 49 spaces.

Each time a bacterium doubles and/or conjugates, we consider that a time step has passed. The total simulated area is equivalent to 1 mm^2^ because we consider that each space can be occupied by only one bacterium, which is approximately 1 μm^2^.

#### 4.1.3. Process Overview and Scheduling

In each cycle:

- We check if there is an empty space in the local neighborhood and if a random number is less than the conjugation rate—in that case, the submodel “bacterial_growth” is activated, and we update one of the empty spaces in the bacterium neighborhood, with the same characteristics as the original bacterium;
- If the bacterium is a donor or a transconjugant, we check if there is a recipient bacterium in the local neighborhood, and if a random number is smaller than the conjugation rate—in that case, the submodel “conjugation” is activated and the recipient bacterium becomes a transconjugant with its characteristics;
- We verify if 95% of the total spaces are filled—in that case, we randomly eliminate bacteria until we have only 50% of the spaces filled.

This process is repeated until the elimination step occurs 1073 times. The information about the amount of each type of bacteria at each time the grid was filled is saved into a .csv file by activating the submodel “files_writing”.

#### 4.1.4. Design Concepts

In this work, we took into consideration these design concepts:

- Basic principles. This model is an adaptation of the model developed in [17]. The major difference is that we consider that the compensatory mutations that reduce the cost of bacteria carrying the plasmid occur in the plasmid rather than the chromosome. Therefore, after a compensatory mutation, the transconjugant bacteria will transfer the plasmid with the already reduced cost. Furthermore, each time the transconjugant bacteria transfers a plasmid, the adaptation time is also already reduced, being equal to that of the transconjugant bacterium that transferred the plasmid. This is because we consider that the plasmid has to be 70 or 400 times in transconjugant bacteria that divide;
- Emergence. The final densities of bacteria will depend on their growth throughout the simulations. In turn, this growth will vary depending on the type of bacteria that are in the nutrient neighborhood and consequently on the competition of those bacteria for resources. The rate of conjugation will also influence the final bacterial densities;
- Adaptation. The number of empty spaces in the nutrient neighborhood divided by the total number of spaces will define whether there is bacterial growth. If there is, the bacterium will grow to an empty position in the local neighborhood. Plasmids of transconjugant bacteria can adapt and reduce the cost to the bacteria carrying them. This adaptation depends on how many duplications the transconjugant bacteria carrying that plasmid already suffered;
- Interaction. Bacteria that have the plasmid (donors or transconjugants) can interact with bacteria that do not have the plasmid (recipients or segregants). This interaction happens when the bacteria are in the same local neighborhood. When there is conjugation from a donor or a non-adapted transconjugant, the bacteria receiving the plasmid have an associated cost. If the plasmid is transferred from a transconjugant that has already had an adaptation, the plasmid is already adapted and has no cost to the recipient bacterium. In addition to these interactions, the model takes into account mediated interactions since it considers that all bacteria compete for nutrients;
- Stochasticity. The model is initiated by the random distribution of bacteria across grid positions. Therefore, at the initial moment of each simulation, bacteria are always in different locations, and for this reason, the interactions between bacteria vary from simulation to simulation. Furthermore, in each cycle, the bacteria to be updated is randomly chosen. In this way, the number of times each bacterium is chosen can vary, making the model asynchronous. Additionally, in the bacterial growth and conjugation submodel, random numbers are obtained (see the “bacterial_growth” and “conjugation” submodels below), which confers stochasticity to the model. Finally, when we simulate bacterial death, we also randomly choose the bacteria that will be eliminated;
- Observation. The observation we take into account in this model is the densities of each type of bacteria whenever the grid reaches 95% capacity.

#### 4.1.5. Initialization

The simulation starts with the random distribution of donor bacteria and recipient bacteria on the grid. Depending on the simulations, bacteria are distributed in the following quantities: 9900 donors and 100 recipients, 5000 donors and 5000 recipients, 100 donors and 9900 recipients, and 10 donors and 9990 recipients.

This distribution is made by activating the submodel “bacteria_distribution” that assigns to each bacteria its characteristics: coordinates, type, plasmid fitness cost, adaptation period, local neighborhood, and nutrient neighborhood.

#### 4.1.6. Input Data

There is no external input of data.

#### 4.1.7. Submodels

1. Bacterial growth

As in the original model [19], we assume that the existence of an empty space in the local vicinity is necessary for bacterial growth. The growth also depends on the respective growth rate proposed by Krone et al. (2007) [21]. Therefore, the growth rate for the analyzed bacterium is given by:

(1) $$\psi (C)=\left\{\begin{array}{c} \psi^{max},\;if\;C\geq \theta \\ \psi^{max}\frac{C}{\theta },\;if\;0\leq C<\theta \end{array}\right.$$

The ψ^*max*^ is 1—c for donor bacteria and transconjugants with the adapted plasmid and 1—b for transconjugants with the non-adapted plasmid. The *C* value is the proportion of empty spaces in the nutrient neighborhood and is obtained by dividing the empty spaces in the nutrient neighborhood by the total number of spaces in that neighborhood (i.e., 7 × 7 = 49 spaces). If there is an empty space in the local neighborhood, we consider that there is bacterial growth if a random number is less than or equal to the growth rate obtained for the bacteria analyzed. In this situation, we update the information of the empty space to the information from the new bacterium. This bacterium will have the characteristics of the original bacterium. If it is a plasmid-bearing bacterium, we will also check for segregation, i.e., loss of the plasmid. Thus, if a random number is less than or equal to the segregation rate, the new bacterium will be of the segregated type and will not have any growth costs. If the bacterium is carrying a non-adapted plasmid, the adaptation time decreases by one. If it reaches zero, the cost is updated to the permanent cost (c). All the parameters are described in Table 1.

2. Conjugation

This process occurs when the selected bacterium is a plasmid-bearing cell (donor or transconjugant). In this submodel, we also follow [17,19]. Therefore, for conjugation to occur, there must be a plasmid-free bacterium present in the local neighborhood. In addition, we calculate the conjugation rate (γ), given by:

(2) $$\gamma \left(C\right)=\left\{\begin{array}{c} \gamma_{max}{}^{}\;\;\;\;if\;C\geq \theta_{2}\\ \frac{C-\theta_{1}}{\theta_{2}-\theta_{1}}\gamma_{max}\;if\;\theta_{1}\leq C<\theta_{2}\\ 0\;\;\;\;\;\;if\;\;\;\;\;C<\theta_{1} \end{array}\right.$$

If there is a recipient or segregating bacterium in the local neighborhood, we consider that there is conjugation if a random number is less than or equal to the conjugation rate obtained for the analyzed bacterium. In this case, we update the information of one of the recipients or segregant bacteria in the neighborhood at random. This bacterium is afterward updated to be of type ‘transconjugant’. The plasmid cost is also updated to c if the plasmid is transferred through a transconjugant with the plasmid already adapted or to b if the plasmid is transferred from a donor or transconjugant with the plasmid not adapted. The adaptation time will be the initial value if the plasmid comes from a donor or the current value of the transconjugant that transferred the plasmid. Note that if the bacterium that receives the plasmid is a segregant bacterium that originated from a donor bacterium, after receiving the plasmid, it will be considered a donor again with cost c.

3. Fitness analysis

The relative success of bacteria (of type A) compared to other bacteria (of type B) cells is obtained by:

(3) SAB=LnNAfNAiNBfNBitime=LnNAfNAi−LnNBfNBitime=mA−mB

Here, $N_{A_{f}}$, $N_{A_{i}}$, $N_{B_{f}}$ and $N_{B_{i}}$, are the final and initial numbers of both types of bacteria. If the ratio between A and B is higher at the end of the simulation relative to the initial ratio, then $S_{AB}$ is positive. If the proportion is lower, $S_{AB}$ is negative.

To compare donors’ success when compensatory mutations occur on plasmids with the original model (compensatory mutation on the chromosome), we calculated the difference in $S_{AB}$ for each case. Values greater than zero for this difference indicate that the compensatory mutation on the plasmid is detrimental to the success of the donors.

We performed the statistical analysis in R v.3.5.1, available at http://www.rstudio.com/ (accessed on 23 February 2023) [38]. We simulated all cases three times and performed one-sample Student *t*-tests (α = 0.05).

## 5. Conclusions

Most bacteria live in structured habitats where social interactions play an important role in microbial evolution [23,24]. After considering this, we have shown here that plasmids play the role of biological weapons of donor cells even if compensatory mutations occur in plasmids rather than in chromosomes. Therefore, rather than dictating plasmid extinction, plasmid cost on transconjugants contribute to maintaining these mobile genetic elements in cells where the plasmid and the chromosome have already co-adapted. Therefore, pathogens adapted to their plasmids, perhaps harboring virulence and resistance genes, are expected to keep circulating without losing their plasmids even if humans would decide to stop using antibiotics.

Future studies must experimentally test this hypothesis, namely that plasmid-bearing cells may use plasmids as biological weapons.

## Figures and Tables

**Table 1:** Parameters of the model.

Entities	Parameter Range	Description
grid_edge	1000	Maximum valeu for x and y coordinates
maximum_proportion_full_grid	0.95	Maximum proportion of bacteria that the grid can contain
remaining_proportion_grid	0.5	Proportion of bacteria remaining in the grid when bacteria are randomly removed when the grid reaches 95% capacity
number_plasmid_free_bacteria	9990, 9900, 5000, 100	Initial number of bacteria not carrying plasmid
donor_bacteria	10, 100, 5000, 9900	Initial number of bacteria that carry plasmid
maximum_growth_rate (ψ^*max*^)	1	Maximum bacterial growth rate
maximum_conjugation_rate (γ*_max_*)	1	Maximum bacterial conjugation rate
theta (*θ*)	0.8	Value of theta (bacterial growth)
theta_1 (𝜃_1_)	0.2	Value of theta 1 (conjugation)
theta_2 (𝜃_2_)	0.3	Value of theta 2 (conjugation)
initial_plasmid_cost (b)	0.2, 0.4, 0.6	Cost that bacterium has when receiving the plasmid
permanent_plasmid_cost (c)	0, 0.1	Cost associated with the presence of the plasmid in donors and adapted transconjugants
adaptation_time	70, 400	Number of duplications that bacteria need until the initial plasmid cost changes to permanent plasmid cost
segregation_probability	0.001	Probability of a bacterium losing the plasmid at the moment of its duplication

## Data Availability

Code and data are available on GitHub (https://github.com/jrebelo27/harmfull_plasmids_compensatory_mutation_in_plasmid, accessed on 25 April 2023).
